# Metformin increases 3-hydroxy medium chain fatty acids in patients with type 2 diabetes: a cross-sectional pharmacometabolomic study

**DOI:** 10.3389/fendo.2024.1313597

**Published:** 2024-02-02

**Authors:** Khaled Naja, Najeha Anwardeen, Ahmed M. Malki, Mohamed A. Elrayess

**Affiliations:** ^1^ Biomedical Research Center, Qatar University, Doha, Qatar; ^2^ Biomedical Science Department, College of Health Sciences, Qatar University (QU) Health, Qatar University, Doha, Qatar

**Keywords:** metformin, metabolomics, 3-hydroxy medium chain fatty acids, type 2 diabetes, anti-diabetic drugs

## Abstract

**Background:**

Metformin is a drug with a long history of providing benefits in diabetes management and beyond. The mechanisms of action of metformin are complex, and continue to be actively debated and investigated. The aim of this study is to identify metabolic signatures associated with metformin treatment, which may explain the pleiotropic mechanisms by which metformin works, and could lead to an improved treatment and expanded use.

**Methods:**

This is a cross-sectional study, in which clinical and metabolomic data for 146 patients with type 2 diabetes were retrieved from Qatar Biobank. Patients were categorized into: Metformin-treated, treatment naïve, and non-metformin treated. Orthogonal partial least square discriminate analysis and linear models were used to analyze differences in the level of metabolites between the metformin treated group with each of the other two groups.

**Results:**

Patients on metformin therapy showed, among other metabolites, a significant increase in 3-hydroxyoctanoate and 3-hydroxydecanoate, which may have substantial effects on metabolism.

**Conclusions:**

This is the first study to report an association between 3-hydroxy medium chain fatty acids with metformin therapy in patients with type 2 diabetes. This opens up new directions towards repurposing metformin by comprehensively understanding the role of these metabolites.

## Graphical Abstract

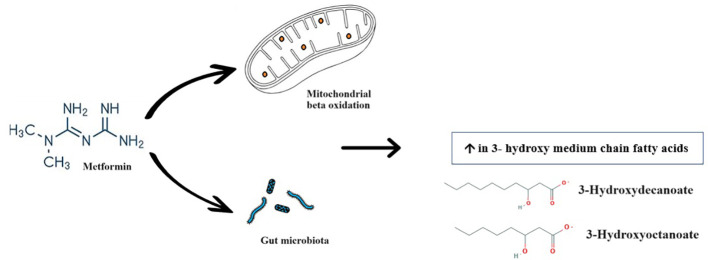

## 1 Introduction

Metformin is undisputedly a milestone in the treatment of type 2 diabetes (T2D). The well-known advantages of metformin include its efficacy and safety profile, success in combination therapy, associated modest body weight reduction, and low cost (1). Although metformin has been utilized for several decades, the complete understanding of its mechanism of action and its effects on metabolism is still lacking. Indeed, the mechanism of action of metformin is complex, and continues to be actively debated and investigated (2). The main mechanisms of metformin are summarized in **Table 1**.

**Table 1:** Main mechanisms of action of metformin on metabolism.

Metabolite/pathway	Mode of action
Hepatic glucose	Metformin reduces the amount of glucose produced by the liver, primarily by inhibiting the mitochondrial respiratory which leads to activation of AMPK (3).
Intestinal glucose	Metformin reduces the absorption of glucose in the intestines and increases the secretion of GLP-1 (4).
Fatty acids	Metformin suppresses fatty acid synthesis mainly by activation of AMPK (5), and increase fatty acid oxidation (6).
Gut microbiome	Metformin alters the gut microbiota composition and function (7, 8).
Cholesterol	Metformin modulates cholesterol synthesis by inhibiting the HMG-CoA reductase enzyme (9).

The repurposing of metformin has been expanded to include many pathophysiological conditions including cancer, cardiovascular diseases, and polycystic ovary syndrome. Moreover, recent studies demonstrated antioxidant and antiaging, renoprotective, and anti-inflammatory and immunomodulatory effects of metformin (2).

Metabolomics involves the comprehensive analysis of metabolites in biological systems, providing insights into complex biochemical pathways and the molecular mechanisms underlying drug effects (10). It has been utilized in various studies to investigate the impacts of drugs on cellular processes, identify potential biomarkers for disease and treatment response, and improve the chemical substance synthesis upstream of later development steps (11). This holistic approach has been particularly useful in understanding the underlying molecular pathways of drug effects, enabling researchers to develop more targeted and effective therapies (11, 12).

Greater insight into the underlying metabolic pathways influenced by metformin can provide a comprehensive understanding of its multifaceted mechanisms of action. This knowledge can pave the way for the development of specialized tools and strategies to enhance metformin therapy in the future. In this a cross-sectional study, our objective is to identify blood metabolites associated with metformin treatment in a cohort of 146 samples from type 2 diabetes patients. We analyzed the metabolic profiles of metformin-treated T2D patients in comparison to drug-naïve T2D patients, and then validated our findings by comparing the profiles of metformin-treated T2D patients with patients treated with other anti-diabetic drugs.

## 2 Methods

### 2.1 Study participants and data source

The study collected data from participants through the Qatar Biobank (QBB), which maintains a comprehensive dataset of Qatari nationals or long-term residents (those residing in Qatar for ≥15 years) aged 18 years and older. The QBB database encompasses various aspects of participant information, including questionnaires that capture details on diabetes history, medication usage, and laboratory results for a wide range of clinically significant metabolic traits. These traits include physical measurements such as blood pressure and body mass index (BMI), clinical chemistry parameters, endocrinology test results, as well as extensive metabolomics data comprising information on over 3000 metabolites. All clinical parameters were measured at the central laboratory of Hamad Medical Corporation in Qatar, accredited by the College of American Pathologists.

Out of the total participants, a cohort of 146 individuals diagnosed with type 2 diabetes, and possessing accessible metabolic data was carefully chosen. The diagnostic criteria for T2D, in accordance with the guidelines set forth by the American Diabetes Association (ADA), were meticulously followed during participant selection (13). Patients with incomplete or inconsistent medication records were excluded from the study. The research was approved by the Institutional Review Boards of the Qatar Biobank (QF-QBB-RES-ACC- 00125). Informed consent was obtained from all participants. Patients were categorized into three groups for this study. The first group consisted of patients who were undergoing treatment with metformin monotherapy, with daily doses ranging from 1000 to 2000 mg. The second group included patients who had not previously received any pharmacological treatment for their condition; these individuals were either newly diagnosed with T2D or had been managing their diabetes solely through lifestyle modifications. The third group comprised patients who were not prescribed metformin, but were instead receiving other anti-diabetic drugs, mainly gliclazide, sitagliptin, or a combination of both.

### 2.2 Metabolomics

All participant serum samples were subjected to untargeted metabolomics using established methods. A detailed explanation of the protocols has already been provided (14). Internal standards and quality checks have been previously published (15). Quality control samples were employed to monitor the stability and consistency of the process over time. A systematic approach was adopted for managing pre-analytical aspects of the samples, such as collection, storage, and preparation, to minimize variability and ensure sample integrity.

### 2.3 Statistical analysis

The metabolomics data were inverse rank normalized. Multivariate analysis including unsupervised (principal component analysis) PCA and supervised (orthogonal partial least square-discriminant analysis) OPLS-DA were conducted using the software SIMCA^®^ (version 18.0.0). Linear models for each metabolite (as the response variable) versus ‘Group 1’ vs ‘Group 2’, then ‘Group 1’ vs ‘Group 3’ (as the explanatory variables) were performed using R (version 4.2.1). The model also included the following confounders: Age, gender, BMI and principal components 1 and 2. The nominal p-values were adjusted using the multiple testing correction method (False Discovery Rate, FDR). Statistical significance was set as FDR < 0.05.

## 3 Results

### 3.1 General characteristics of participants

**Table 2** provides an overview of the general characteristics of participants in this study, grouped by their treatment regimens for T2D. Notably, several parameters exhibited significant differences among the groups. Gender distribution differed significantly between the metformin-treated group (G1) and Drug-naïve (G2) group, while age was notably distinct in (G1) compared to (G2). Metformin-treated individuals (G1) showed significant differences in insulin levels and homeostatic model assessment of insulin resistance (HOMA-IR) when compared to the non-metformin-treated (G3) group. Additionally, variations were observed in total cholesterol (TC), LDL cholesterol, systolic blood pressure, pulse rate, albumin, alkaline phosphatase, total iron, Free triiodothyronine, ferritin, total dihydroxyvitamin D, and other parameters across different groups.

**Table 2:** General characteristics of participants.

	Metformin- treated T2D (Group 1)	Drug-naïve T2D (Group 2)	Non-metformin- treated T2D (Group 3)	G1 vs G2 p-value	G1 vs G3 p-value
N	54	47	45		
Gender					
Male	27 (50%)	36 (76.6%)	27 (60%)	0.011	0.428
Female	27 (50%)	11 (23.4%)	18 (40%)		
Age	50.07 (9.18)	44.3 (10.11)	48.51 (9.93)	0.004	0.422
Body mass index (kg/m^2^)	31.62 (27.26-34.56)	30.35 (27.34-33.41)	31.62 (28.31-34.94)	0.494	0.471
Waist-hip ratio	0.89 (0.1)	0.92 (0.08)	0.9 (0.1)	0.187	0.709
Fasting blood glucose (mmol/L)	7.45 (6.2-9.38)	7.05 (6.23-8.95)	8.2 (7-13.3)	0.726	0.071
HbA1c (%)	6.4 (5.9-7.7)	6.6 (5.9-7.8)	6.9 (6-8.2)	0.642	0.186
Insulin (uU/mL)	11.8 (8.27-23)	16 (8-50.4)	22.8 (11.6-66.1)	0.240	0.007
C-Peptide	2.66 (1.77-3.86)	3.38 (2.46-6.86)	3.51 (2.18-6.74)	0.014	0.066
HOMA-IR	3.66 (2.55-7.81)	5.21 (2.7-21.63)	10.29 (4.14-30.13)	0.184	0.001
TC (mmol/L)	5 (0.92)	5.22 (0.83)	5 (4.4-5.67)	0.210	0.691
HDL (mmol/L)	1.25 (1.04-1.44)	1.1 (0.99-1.25)	1.16 (1.02-1.38)	0.107	0.560
LDL (mmol/L)	2.87 (0.91)	3.26 (0.77)	3.06 (0.98)	0.022	0.316
TG (mmol/L)	1.6 (1.13-2.31)	1.6 (1.11-2.06)	1.73 (1.2-2.4)	0.612	0.770
Systolic blood pressure	121 (111.25-129.75)	120 (113-130.75)	122 (115–135)	0.753	0.191
Diastolic blood pressure	76 (68.25-80)	81 (74.5-86.75)	79 (72–84)	0.001	0.080
Pulse rate	71 (62–78)	71.5 (65.25-79)	76 (70–83)	0.502	0.033
Albumin (g/L)	44.48 (2.63)	45.06 (2.94)	43.27 (2.87)	0.299	0.032
Alkaline Phosphatase (U/L)	67.5 (61-77.5)	68 (55–79)	75 (64–88)	0.560	0.036
ALT (U/L)	21.5 (16–29)	23 (17.5-31.5)	20 (16–29)	0.359	0.683
AST (U/L)	17 (15.25-21)	17 (15–24)	16 (14–21)	0.806	0.470
Bicarbonate (mmol/L)	26.3 (2.49)	26.53 (2.54)	25.93 (2.67)	0.640	0.490
Total Bilirubin (µmol/L)	6 (4.03-7.77)	6.8 (4.65-8.88)	6 (5-8.8)	0.118	0.366
Calcium (mmol/L)	2.38 (0.09)	2.39 (0.08)	2.37 (0.08)	0.408	0.785
Chloride (mmol/L)	100.44 (1.87)	100.53 (2.19)	100 (98–102)	0.831	0.165
CRP (mg/L)	6 (5–8)	5 (5–7)	7 (5-11.25)	0.173	0.257
Creatine Kinase (U/L)	76 (53-126)	108 (62-148)	92.5 (62-126.75)	0.233	0.624
Creatine Kinase-1 (ng/ml)	1.08 (0.89)	1.56 (0.49)	1.96 (1.04)	0.457	0.283
Creatinine (µmol/L)	68.5 (54-79)	74 (64-79)	66 (52-76)	0.102	0.435
Total Dihydroxyvitamin D (ng/ml)	L20 (14-26)	15 (12-20)	17 (13.75-23)	0.015	0.163
Estradiol (pmol/L)	78.5 (56.25-110)	98 (69.5-129.5)	89.5 (64-140)	0.103	0.275
Ferritin (µg/L)	53.5 (24-122)	98 (61-179)	84 (26-166)	0.004	0.210
Free Thyroxine (pmol/L)	12.87 (12.12-14.28)	12.9 (12.47-13.72)	13.13 (1.81)	0.685	0.909
Free Triiodothyronine (pmol/L)	4.33 (3.91-4.62)	4.6 (4.2-4.9)	4.31 (0.56)	0.014	0.794
GGT (U/L)	22.5 (14.5-25)	27 (23-40)	24 (16.75-34.25)	0.039	0.429
Iron (µmol/L)	14.56 (4.78)	16.8 (5.38)	14.45 (5.45)	0.015	0.916
Magnesium (mmol/L)	0.79 (0.06)	0.83 (0.07)	0.8 (0.06)	0.006	0.550
Phosphorus (mmol/L)	1.11 (0.17)	1.09 (0.21)	1.1 (0.21)	0.503	0.679
Potassium (mmol/L)	4.3 (4.2-4.5)	4.3 (4.1-4.55)	4.2 (4.1-4.5)	0.673	0.385
Sodium (mmol/L)	139.76 (2.14)	139.98 (2.12)	140 (138-141)	0.645	0.621
Testosterone Total (nmol/L)	5.04 (0.92-14.3)	14.52 (6.44-17.24)	9.75 (0.85-16.26)	0.005	0.610
TSH (mIU/L)	1.41 (1.1-1.97)	1.18 (0.96-1.9)	1.18 (0.82-1.82)	0.269	0.197
Total Protein (g/L)	71.83 (3.91)	72.98 (3.76)	71.91 (3.6)	0.137	0.918
Urea (mmol/L)	4.37 (1.15)	4.58 (0.94)	4.5 (3.8-5.1)	0.318	0.154
Uric Acid (µmol/L)	304.69 (68.89)	325.64 (86.69)	290.04 (88.77)	0.187	0.369

Data are presented as mean (SD), median (IQR), and number for parametric, non-parametric, and nominal variables, respectively. The difference between the mean/median was evaluated using an independent t-test/Mann–Whitney U test. The chi-square test was used for the nominal variable.

### 3.2 Multivariate analysis of metabolites differentiating between the treatment groups

**Figure 1:**
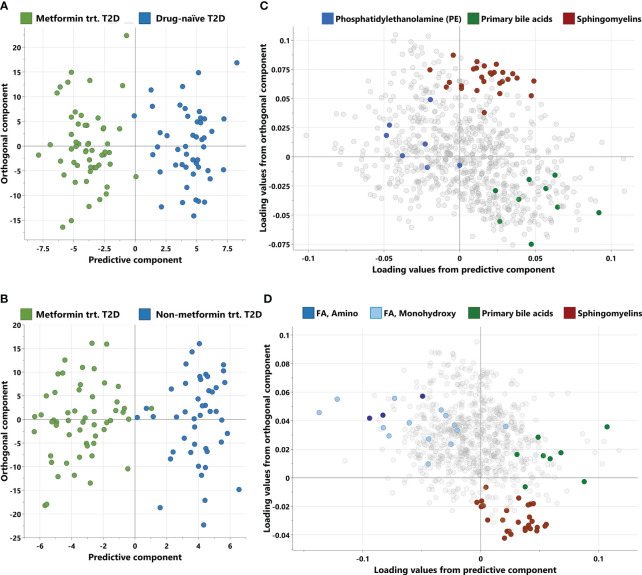
Score and loading plots from OPLS-DA. **(A, B)** Score plots showing predictive component (x-axis) and orthogonal component (y-axis) between metformin-treated T2D vs drug-naïve T2D and metformin treated T2D vs non-metformin treated T2D respectively. **(C, D)** Corresponding loading plots depicting the enriched pathways for each OPLS-DA respectively. trt, treated; FA, Fatty acid.

Non-targeted metabolomics analysis was conducted to explore the metabolic profiles of 146 T2D patients, classified into three distinct groups. The OPLS-DA method, depicted in **Figure 1**, was employed to identify key discriminating components between metformin-treated individuals (Group 1) and drug-naïve patients (Group 2), as well as between Group 1 and non-metformin-treated patients (Group 3). Scatter plots in **Figures 1A, B** clearly exhibit the distinct separation of group 1 from group 2, and group 1 from group 3, respectively. Furthermore, **Figures 1C, D** display the corresponding loading plots, revealing the primary metabolic pathways responsible for distinguishing group 1 from group 2, and group 1 from group 3, respectively. These include phosphatidylethanolamine, primary bile acids and sphingomyelins for group 1 vs group 2, and amino fatty acid, monohydroxy FA, primary bile acids and sphingomyelins for group 1 vs group 3.

### 3.3 Univariate analysis of metabolites differentiating group 1 and 2

The linear model analysis revealed several significant changes with false discovery rate (FDR) adjustments between the metformin-treated group and the drug-naïve group, as illustrated in **Table 3**. Notably, the metformin-treated group exhibited an increase in 3-hydroxydecanoate, 3-hydroxyoctanoate, and ethyl alpha-glucopyranoside. Conversely, a decrease was observed in citrulline, gamma-glutamylcitrulline, N6,N6,N6-trimethyllysine, as well as various sphingomyelins.

**Table 3:** Metabolites differentiating metformin-treated T2D vs drug-naïve T2D after correcting for age, gender, BMI and principal components 1 and 2.

Metabolites	Super-pathway	Sub-pathway	Estimate	SE	p-value	FDR
3-hydroxydecanoate	Lipid	Fatty Acid, Monohydroxy	0.909	0.197	1.42 x 10^-5^	0.012
Sphingomyelin (d18:2/18:1) *	Lipid	Sphingomyelins	-0.720	0.170	5.69 x 10^-5^	0.017
N6,N6,N6-trimethyllysine	Amino Acid	Lysine Metabolism	-0.695	0.174	1.33 x 10^-4^	0.017
3-hydroxyoctanoate	Lipid	Fatty Acid, Monohydroxy	0.840	0.210	1.37 x 10^-4^	0.017
Citrulline	Amino Acid	Urea cycle; Arginine and Proline Metabolism	-0.922	0.232	1.42 x 10^-4^	0.017
Taurochenodeoxycholic acid 3-sulfate	Lipid	Secondary Bile Acid Metabolism	-0.860	0.214	1.44 x 10^-4^	0.017
N-acetyltyrosine	Amino Acid	Tyrosine Metabolism	-0.705	0.178	1.45 x 10^^-4^	0.017
Ethyl alpha-glucopyranoside	Xenobiotics	Food Component/Plant	1.720	0.422	4.05 x 10^-4^	0.038
Sphingomyelin (d18:1/20:2, d18:2/20:1, d16:1/22:2)*	Lipid	Sphingomyelins	-0.715	0.195	4.14 x 10^-4^	0.038
Gamma-glutamylcitrulline*	Peptide	Gamma-glutamyl Amino Acid	-0.773	0.215	5.45 x 10^-4^	0.040
Sphingomyelin (d18:2/24:2)*	Lipid	Sphingomyelins	-0.608	0.169	5.46 x 10^-4^	0.040
Sphingomyelin (d18:2/24:1, d18:1/24:2)*	Lipid	Sphingomyelins	-0.654	0.183	5.89 x 10^-4^	0.040

(*) Indicates that Metabolon is confident about the identity of this metabolite, although it has not been officially confirmed based on a standard.

### 3.4 Univariate analysis of metabolites differentiating group 1 and 3

The linear model analysis also revealed several significant changes with FDR adjustments between the metformin-treated group and the non-metformin-treated group, as presented in **Table 4**. Notably, the metformin-treated group exhibited an increase in 3-hydroxydecanoate, 3-hydroxyoctanoate, betonicine, and 2-aminooctanoate. Conversely, a decrease was observed in citrulline, gamma-glutamylcitrulline, as well as various secondary bile acid metabolites.

**Table 4:** Metabolites differentiating metformin treated T2D vs non-metformin treated T2D after correcting for age, gender, BMI and principal components 1 and 2.

Metabolites	Super-pathway	Sub-pathway	Estimate	SE	p-value	FDR
3-hydroxydecanoate	Lipid	Fatty Acid, Monohydroxy	0.986	0.194	1.89 x 10^-6^	1.55 x 10^-3^
Glycochenodeoxycholate 3-sulfate	Lipid	Primary Bile Acid Metabolism	-0.826	0.184	2.04 x 10^-5^	5.14 x 10^-3^
Taurochenodeoxycholic acid 3-sulfate	Lipid	Secondary Bile Acid Metabolism	-0.834	0.185	2.14 x 10^-5^	5.14 x 10^-3^
3-hydroxyoctanoate	Lipid	Fatty Acid, Monohydroxy	0.892	0.201	2.51 x 10^-5^	5.14 x 10^-3^
Glycodeoxycholate 3-sulfate	Lipid	Secondary Bile Acid Metabolism	-0.731	0.174	6.55 x 10^-4^	0.011
Gamma-glutamylcitrulline*	Peptide	Gamma-glutamyl Amino Acid	-0.841	0.211	1.32 x 10^-4^	0.018
Citrulline	Amino Acid	Urea cycle; Arginine and Proline Metabolism	-0.868	0.227	2.42 x 10^-4^	0.028
Betonicine	Xenobiotics	Food Component/Plant	0.838	0.218	3.13 x 10^-4^	0.031
Taurodeoxycholic acid 3-sulfate	Lipid	Secondary Bile Acid Metabolism	-0.694	0.185	3.45 x 10^-4^	0.031
2-aminooctanoate	Lipid	Fatty Acid, Amino	0.656	0.178	4.00 x 10^-4^	0.033

(*) Indicates that Metabolon is confident about the identity of this metabolite, although it has not been officially confirmed based on a standard.

### 3.5 Univariate analysis common metabolites

**Figure 2:**
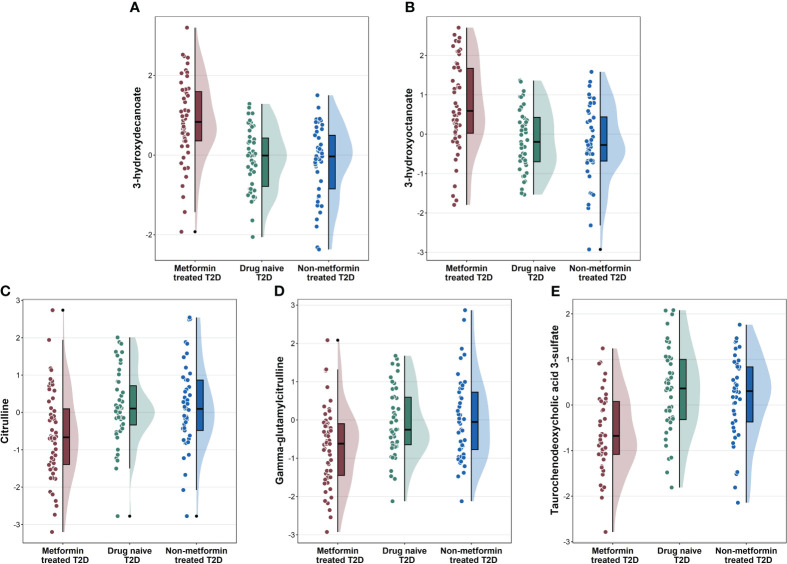
Raincloud plots showing the novel metabolites **(A, B)**, and other metabolites **(C–E)** associated with metformin-treated T2D individuals compared to drug-naïve T2D and non-metformin treated T2D from the linear regression analysis.

To identify metabolite changes specifically associated with metformin treatment, we analyzed the common metabolites that significantly differentiated between group 1 vs. group 2 and group 1 vs. group 3 (**Figure 2**). These changes included higher levels of 3-hydroxydecanoate, and 3-hydroxyoctanoate (**Figures 2A, B**) in group 1 compared to groups 2 and 3, and lower levels of citrulline, gamma-glutamylcitrulline, and taurochenodeoxycholic acid 3-sulfate (**Figures 2C–E**) in group 1 compared to groups 2 and 3.

## 4 Discussion

The pathogenesis of T2D is characterized by a complex interplay of various metabolic abnormalities, including reduced peripheral glucose utilization, impaired beta-cell function, increased lipolysis, elevated free fatty acid levels, and gut dysbiosis (16). Obesity, ethnicity, physical activity, and socioeconomic status are risk factors that contribute to the development and progression of T2D (17). Metformin is currently the first-line medication to treat T2D in most guidelines. The mechanisms underlying the therapeutic action of metformin are complex and are still not fully understood (2). Pharmacometabolomics is a research field aimed at achieving a comprehensive and systemic understanding of drug mechanisms.

In this study, we employed an untargeted metabolomics approach to investigate the metabolic changes associated with metformin treatment in patients with T2D. Our emerging data revealed two novel metabolites, namely 3-hydroxyoctanoate and 3-hydroxydecanoate, which have not been previously reported in association with metformin treatment. While previous studies have reported lipid alterations linked to metformin treatment (18, 19), our findings are in line with earlier research (20, 21), demonstrating that citrulline and gamma-glutamylcitrulline are associated with metformin intake. The observed increase in 3-hydroxyoctanoate and 3-hydroxydecanoate levels in the metformin-treated group, when compared to the drug-naïve group, was further supported by comparisons with the non-metformin treated group. These metabolites will be the primary focus of our discussion.

### 4.1 3-hydroxy medium chain fatty acids metabolites

3-hydroxyoctanoate and 3-hydroxydecanoate are primary metabolites that belong to the class of organic compounds known as 3-hydroxy medium-chain fatty acid (MCFA). These are hydroxy saturated fatty acids with 6 to 12 carbon atoms long side chain. MCFAs are natural compounds present in both animal and plant tissues that participate in cell metabolism. Like short-chain fatty acids, MCFAs play an important role in intracellular signaling and contribute to the regulation of cell metabolism. MCFAs have metabolic properties that are distinct from those of long-chain fatty acids, which make them readily oxidized by cells, provide a very efficient source of energy production, and impact postprandial energy expenditure positively (22). 3-hydroxy MCFAs are intermediates of mitochondrial β-oxidation (23), and of microbial origin (24).

### 4.2 Mitochondrial beta oxidation

Mitochondria play a pivotal role in the pharmacodynamics of metformin (25). Metformin promotes mitochondrial fatty acid oxidation (26, 27). It activates AMP-activated protein kinase (AMPK) which reduces the activity of Acetyl-CoA carboxylase 1 and 2 (ACC1/2), thus reducing malonyl-CoA levels, leading to an increase in fatty acid oxidation in the mitochondria (19). Metformin also leads to an increase in the expression of peroxisome proliferator-activated receptor gamma coactivator 1-alpha (PGC1-α) in various cell types, including hepatocytes and skeletal muscle cells (28). PGC1-α stimulates fatty acid targeting for mitochondrial β-oxidation thus diminishing the rate of fatty acid storage, the synthesis of lipotoxic derivatives, and triacylglycerol storage and secretion (29).

In line with our results, Walcott et al. reported a metabolic signature of increased fatty acid beta-oxidation and 3-hydroxy fatty acids among Li-Fraumeni syndrome (LFS) patients upon metformin treatment (6). However, our emerging data showed only the increase of 3-hydroxy medium chain fatty acids in the metformin-treated group when compared to each of the two other groups. No significant changes in other beta oxidation intermediates were shown. This suggests that the rate of beta oxidation was reduced at a certain step that favors the accumulation of these particular metabolites. This is consistent with many studies that reported the production of 3-hydroxy fatty as a result of incomplete beta-oxidation (30, 31).

In fact, the enzyme 3-hydroxyacyl-CoA dehydrogenase catalyzes the penultimate reaction of the mitochondrial β-oxidation cascade, the NAD^+^ dependent conversion of L-3-hydroxyacyl-CoA to 3-ketoacyl-CoA (32). No medium-chain 3-L-hydroxyacyl-coA dehydrogenase has been identified in humans; however, the human mitochondrial trifunctional protein (MTP) complex that possesses 3-hydroxyacyl-coA dehydrogenase activity is active with substrates of acyl-chain length from C6 to C16 (33). Interestingly, 3-hydroxyacyl-coA dehydrogenase donates electrons directly to complex I of the electron transport chain (ETC) (34); moreover, the MTP complex has been demonstrated to interact with the NADH-binding domain of complex I of the ETC (35), yet the exact nature of this relationship and its implications for cellular metabolism are not yet fully understood. Taking into consideration that metformin inhibits the complex I of ETC (36), the inhibition of this complex may lead to a decrease in the activity of 3-hydroxyacyl-coA dehydrogenase, and the increase in the levels of 3-hydroxy medium-chain fatty acids.

Another plausible explanation is through the mitochondrial enzyme deacetylase Sirtuin 3 (SIRT3). SIRT3 is essential for maintaining mitochondrial function, and it has been verified to regulate aging, neurodegeneration, liver and heart diseases, and other metabolic diseases (37). Sirt3 was shown to mediate, at least partly, the metformin-induced AMPK activation and cardioprotection (3). SIRT3 regulates fatty acid oxidation (38) via enzyme deacetylation. Interestingly, 3-hydroxyacyl-CoA dehydrogenase is a highly probable substrate for SIRT3 deacetylation (39). On the other hand, metformin reduces hepatic expression of SIRT3 and mitochondrial SIRT3 protein levels (40). This reduction of SIRT3, could ultimately lead to the inhibition of 3-hydroxyacyl-CoA dehydrogenase, and increase in the 3-hydroxy medium chain fatty acids.

Intriguingly, SIRT3 directly deacetylates and regulates the activity of ornithine transcarbamoylase (OTC), the enzyme that catalyzes the formation of citrulline from ornithine (39). The reduction of SIRT3 activity by metformin and subsequent inhibition of ornithine transcarbamoylase could explain the significant decrease in the levels of citrulline and its metabolite gamma-glutamylcitrulline in the metformin-treated group when compared to each of the two other groups. Nevertheless, it is undeniable that more future research studies are still needed to fully elucidate the interaction mechanism between mitochondria and metformin.

### 4.3 Gut microbiota-derived metabolites

Microbial metabolism could be one of the most relevant source of 3-hydroxy medium chain fatty acids (41). Sjögren et al. reported the production of several 3-hydroxy fatty acids, including 3-hydroxydecanoic acid, from *Lactobacillus plantarum* (42), which belongs to the phylum Firmicutes, one of the two major phyla that dominate the gut microbiota (43). Moreover, Le Roy et al. showed that yogurt consumption was associated with reduced visceral fat mass, changes in gut microbiome, and importantly, an increase of 3-hydroxyoctanoic acid among other metabolites (44). However, according to Mikkelsen et al., 3-hydroxydecanoate is not produced by the gut microbiota, but it may still regulate its levels (45). The crosstalk between the metabolism of gut microbiota and metformin is now well ingrained (46). Increasing the production of short-chain fatty acids is among the mechanisms by which metformin exerts part of its hypoglycemic effects via the gut microbiota (7, 47). Though more validation studies are needed, we hypothesize that metformin-induced gut microbiota metabolites can also include 3-hydroxy MCFAs.

### 4.4 Effects of 3-hydroxy MCFAs

3-hydroxy MCFAs act as agonists of the hydroxycarboxylic acid receptor 3 (HCA_3_), also known as GPR109B (48). HCA_3_ is a G-protein-coupled receptor (also called metabolite-sensing G protein-coupled receptor), which regulates metabolism and immune functions (49). It is exclusively expressed in humans and higher primates (23), and this is presumably the reason why the receptor is still insufficiently studied because of lack of accessible animal models. In humans, HCA_3_ is expressed in adipocytes, immune cells, and intestinal epithelium; however, its expression has not been studied in-depth (50). Compared with other endogenous ligands which have low potency, 3-hydroxyoctanoic acid is highly specific to HCA_3_ (51), and activates it at micromolar concentrations (52). 3-hydroxy MCFAs act also as agonists to G-protein coupled receptor 84 (GPR84) which is primarily expressed in myeloid cells, and has remarkably increased expression in adipocytes when encountering acute inflammatory stimuli (53), and by hyperglycemia and hypercholesterolemia (54). 3-hydroxydecanoic acid is a common agonist of HCA_3_ and GPR84 (49). Strikingly, both HCA_3_ and GPR84 couple to G proteins resulting in the inhibition of adenylyl cyclase, and subsequent decrease of intracellular cyclic AMP (cAMP) levels (49), consistent with the ability of metformin to reduce cAMP levels resulting in decreased cAMP-dependent protein kinase (PKA) activity and reduced phosphorylation of critical PKA target proteins, and ultimate suppression of glucagon-dependent glucose output from hepatocytes (55).

Ahmed et al. demonstrated that 3-hydroxyoctanoic acid exerts an anti-lipolytic activity via HCA_3_ in situations of increased fatty acid oxidation and inhibits free fatty acid release (51). This is likely, at least in part, to explain the observed reduction in FFA after metformin treatment. Noteworthily, a partial inhibition of lipolysis in adipocytes may improve insulin sensitivity (56). The improvement of insulin sensitivity is considered a consequence of the changes induced by metformin in lipid metabolism (19). This corroborates our emerging data showing a significant reduction in HOMA-IR in metformin-treated group when compared to the two other groups, indicating an improvement in insulin sensitivity along with the increase in 3-hydroxy MCFAs.

Additionally, Stäubert et al. provided evidence that HCA_3_ is essential for fatty acid metabolism control in breast cancer cells (57). Moreover, HCA_3_ is an important regulator of inflammation in adipose tissue and may potentially be a target for the treatment of metabolic disorders (30). HCA_3_ activation inhibits the production of reactive oxygen species, and increases anti-inflammatory interleukin 10 secretion (58). Activation of HCA_3_ reduced the production of proinflammatory cytokines in adipocytes and macrophages (59). This is consistent with the anti-oxidative and anti-inflammatory effects of metformin. On the other hand, Recio et al. showed that GPR84 activation triggered increased phagocytosis in macrophages (54). Relatedly, studies reported metformin-induced shift in myeloid cells from classical to nonclassical monocytes, and increased phagocytosis activity (60). Promoting phagocytosis of macrophages is one of anti-tumor strategies (61), and metformin is a very promising drug to treat cancer (62). However, whether metformin promotes phagocytosis by indirect activation of GPR84 through 3-hydroxy MCFA is to be confirmed by in-depth future studies. Interestingly, Peters et al. (63) demonstrated that metabolites of lactic acid bacteria, permanently colonizing the human intestine, are highly potent agonists of HCA_3_. Additionally, Sakura et al. reported the production of HCA_3_ ligands *in vitro* by *Bifidobacterium*, which is one of the major components of the human gut microbiota (64).

Taken together, our results provide evidence that metformin increases the production of 3-hydroxy fatty acids via beta oxidation and/or gut microbiota. Subsequently, these metabolites have the potential to activate the metabolite-sensing G protein-coupled receptors, and thus mediating the pleiotropic beneficial effects of metformin. Although not statistically significant, the metformin-treated group showed better glycemic control and lipid profile compared to the two other groups, suggesting an underlying beneficial role of 3-hydroxy medium chain fatty acids. Yet, other studies hold a conclusion against our findings. Mikkelsen et al. (45) reported that 3-hydroxydecanoate is enriched in the circulation of obese individuals with T2D compared with healthy controls. Al-Sulaiti et al. (65) showed that 3-hydroxyoctanoate and 3-hydroxydecanoate were elevated in patients with T2D when compared to obese insulin resistant and obese insulin sensitive participants. However, these two studies didn’t take into consideration the treatment of the patients with diabetes. Very probably, a significant number of these patients could be on metformin therapy, and the elevation of 3-hydroxydecanoate and/or 3-hydroxydecanoate could be beneficial and attributed to metformin treatment rather than the diabetes condition.

## 5 Conclusion

To our knowledge, we report for the first time an association between 3-hydroxyoctanoate and 3-hydroxydecanoate with metformin treatment in patients with T2D. This could provide insights into the underlying mechanisms of metformin, and help to identify new therapeutic applications of this drug. 3-hydroxy medium chain fatty acids can originate from an increased but incomplete mitochondrial beta oxidation, or produced by the gut microbiota.3-hydroxyoctanoate and 3-hydroxydecanoate are activators of two important receptors, namely hydroxycarboxylic acid receptor 3 and G-protein coupled receptor 84.

While our results were replicated when comparing metformin-treated group with each of the two other groups, we acknowledge that the small sample size is a limitation in this study. Another limitation is the cross-sectional design of the study, which does not allow for the assessment of changes of these metabolites over time. Future research could involve larger cohorts of patients, including different ethnic groups and populations, to validate the findings and explore any potential differences in metabolic signatures.

## Data availability statement

Qatar biobanks owns the intellectual property rights of the data. Requests to access these datasets should be directed to takepart@qatarbiobank.org.qa.

## Ethics statement

The studies involving humans were approved by Institutional Review Boards of the Qatar Biobank (QF-QBB-RES-ACC-00125). The studies were conducted in accordance with the local legislation and institutional requirements. The participants provided their written informed consent to participate in this study.

## Author contributions

KN: Writing – original draft. NA: Software, Writing – original draft. AM: Writing – review & editing. ME: Funding acquisition, Project administration, Supervision, Writing – review & editing.
